# Trends and Developments in Vulvar Lichen Sclerosus Research

**DOI:** 10.1002/hsr2.71007

**Published:** 2025-07-09

**Authors:** Fan‐Rong He, Yan‐Rui Lin, Yu‐Hua Wen, Jin‐He Deng

**Affiliations:** ^1^ Department of Obstetrics and Gynecology The Air Force Hospital of Southern Theater Command Guangzhou China; ^2^ Department of Anesthesiology The Second Affiliated Hospital of Guangzhou University of Chinese Medicine Guangzhou China

**Keywords:** bibliometric, hotspots, topical corticosteroids, trends, vulvar lichen sclerosus

## Abstract

**Background:**

Despite the prevalence of vulvar lichen sclerosus (VLS) as a chronic skin disease in clinical settings, there is a notable absence of a comprehensive bibliometric analysis summarizing the existing literature on this topic. The aim of this study is to offer clinicians, researchers, and other interested parties an up‐to‐date overview of the research status and emerging trends in VLS through the use of bibliometric analysis.

**Methods:**

The present research employed a thorough examination of the Web of Science Core Collection to identify publications pertaining to VLS within the time frame of January 1, 1994, to December 31, 2023. A total of 1698 publications were scrutinized utilizing Microsoft Excel software and various visualization tools, including CiteSpace, VOSviewer, and Pajek, to discern specific characteristics.

**Results:**

The current research encompassed a total of 1,698 articles pertaining to VLS. The analysis of citation bursts and co‐citation patterns has revealed a high level of confidence in the effectiveness and safety of topical corticosteroids for the treatment of VLS, establishing them as the preferred primary treatment for this condition. Since 2018, there has been a significant increase in VLS publications, with the United States and United Kingdom emerging as leading contributors. Research has moved from examining past pathological changes like “epidermal atrophy” to focusing on “long‐term treatment management,” and improving “quality of life”. The exact mechanism of VLS remains unclear, but it is linked to cytokine immune regulation, oxidative stress, and potential epigenetic changes or genetic mutations in susceptible individuals. It could also develop into vulvar squamous cell carcinoma.

**Conclusions:**

Institutions are expected to allocate more resources for VLS prevention and long‐term management.

## 1 Introduction

Vulvar lichen sclerosus (VLS) is a chronic skin condition that causes inflammation, thinning of the skin, and changes in the dermis. It can lead to hypopigmentation, atrophy, and fissuring in the genital area, along with intense itching or pain. Lichen sclerosus (LS) has a global prevalence of 0.1%–1.7% and can affect various body parts, including the genital area of the opposite sex [1, 2, 3]. VLS is a common condition treated in vulvar clinics, but its true prevalence is unknown. Estimates of the prevalence of VLS vary, with reported rates ranging from 1 in 30 older adult females to 1 in 59 females in a general gynecology practice, and 1 in 300–1000 patients referred to dermatologists [4, 5, 6, 7, 8]. Left untreated, VLS is associated with a 4%–6% risk of malignant transformation [9]. There is evidence to suggest that the incidence of VLS may be increasing [10, 11]. VLS is also linked to an elevated risk of vulvar squamous cell carcinoma (SCC), with estimates indicating a risk of less than 5% [12, 13]. The identification of cases of LS with a high probability of progressing to SCC is an active area of research [14].

The etiology of VLS remains unclear and is believed to involve multiple factors, including genetic predisposition, immunological abnormalities, hormonal influences, infections, cell kinetics, and environmental triggers [15, 16, 17, 18]. Diagnosis of VLS typically relies on identifying distinctive clinical features, such as white, atrophic plaques accompanied by fissures, ecchymoses, and anatomical alterations. In cases where confirmation is needed, a biopsy and histopathological analysis can be conducted. The primary focus of treatment should be directed towards the management of inflammation and enhancement of structural changes. Initial therapy generally includes super‐potent topical corticosteroids (TCSs) like clobetasol propionate, administered for 6–12 weeks based on evidence‐based practice, followed by maintenance 2–3 times per week [1, 2, 19, 20]. Nevertheless, there remains ongoing uncertainty regarding the etiology, histological diagnosis, and optimal treatment strategies for this condition.

Despite growing interest in VLS, there is a lack of comprehensive studies on global research activity in this area. The diversity of publications complicates researchers' ability to evaluate their work's academic value and keep abreast of recent developments. Additionally, there is a noticeable absence of a comprehensive bibliometric analysis in this field. Hence, the primary objective of this study is to perform an extensive bibliometric analysis of research articles pertaining to VLS, with the purpose of identifying prominent research areas, potential patterns, and offering recommendations for future research endeavors.

## 2 Methods

### 2.1 Search Strategy

The current investigation utilized a thorough search within the Web of Science Core Collection (WoSCC) by employing the search term “Vulvar Lichen Sclerosus” to compile literature pertaining to VLS. Additionally, the Social Science Citation Index (SSCI) and Science Citation Index Expanded (SCI‐E) were consulted to identify pertinent articles published up to December 31, 2023. The search encompassed the title, abstract, author keywords, and KeyWords Plus fields. Graphical analysis of collaboration incorporated bibliographic coupling, co‐citation, citation, coauthorship, and co‐occurrence metrics. The research concentrated on articles categorized as “Articles” released in English between 1994 and 2023, as outlined in Supporting Information S1: Table S1. The impact factor for each journal was calculated using the 2023 Journal Citation Reports (JCR) data set.

### 2.2 Literature Selection

**Figure 1:**
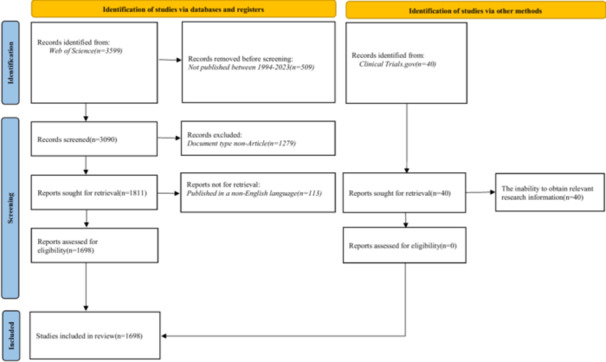
Flow diagram.

A total of 3599 original articles were retrieved from the WoSCC using the search terms: “Lichen sclerosis,” “Lichen sclerosus,” “Lichen sclerosus et atrophicus,” “Vulvar dermatoses,” “Vulvar lichen sclerosus,” and “Vulval lichen sclerosus.” The inclusion criteria for this study were as follows: (1) articles published between 1994 and 2023, (2) documents classified as original articles, and (3) articles written in English. Following screening, 1698 articles were deemed eligible for inclusion based on predetermined criteria, while 1901 articles were excluded due to reasons such as publication outside the specified time frame (*n* = 509), classification as a document other than an “Article” (*n* = 1279), and non‐English language (*n* = 113) (Figure 1).

### 2.3 Bibliometric Analysis

A total of 1698 articles were retrieved from the Web of Science (WoS) platform, with pertinent details such as authors, journals, affiliations, countries, and keywords being extracted for further analysis. These data underwent processing and examination through a range of software tools, including CiteSpace (version 6.1.R6, developed by Chaomei Chen), VOSviewer (version 1.6.18), Pajek (version 5.17), and Microsoft Excel (2021). CiteSpace was employed to pinpoint keywords and references exhibiting notable citation bursts. Moreover, it generates a dual‐map overlay that effectively emphasizes the relevant journals in the field of VLS [21]. Meanwhile, VOSviewer is a commonly employed tool that enables the examination of collaborative networks among countries, institutions, authors, and journals, and visually represents the findings [22]. Recently, bibliometric analyses of skin diseases have garnered increasing attention, with a focus on elucidating the primary research hotspots and emerging frontiers within the field [23, 24, 25, 26]. The tables in this study are derived from WoS, while the figures are sourced from CiteSpace and VOSviewer.

## 3 Results

### 3.1 Publication and Countries Analysis

**Figure 2:**
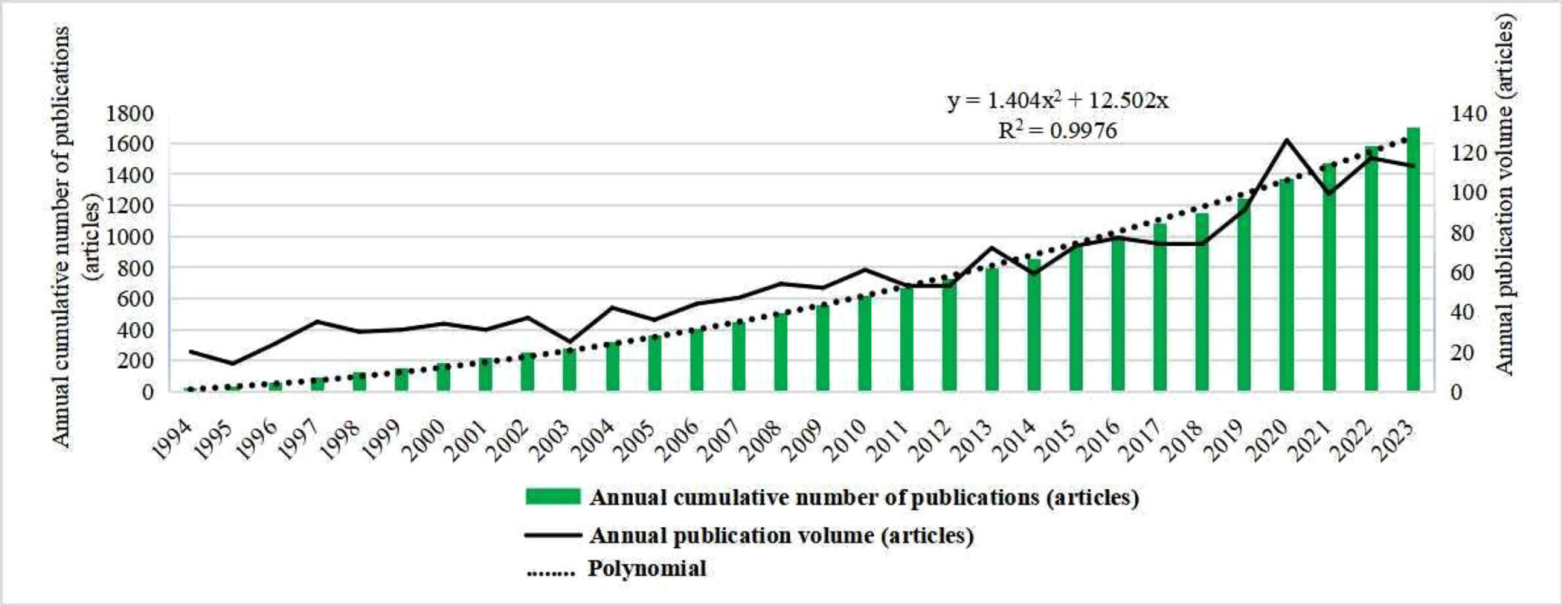
Publication volume.

Between 1994 and 2023, the volume of publications and citations within the field of VLS exhibited a gradual upward trend. Notably, a marked escalation in the number of publications commenced in 2018, signifying an intensified interest among researchers (Figure 2). The United States (USA) emerged as the most prolific contributor to VLS‐related research, with 485 articles published. The notable citation counts and H‐index values attained by both the USA and the United Kingdom (UK) underscore their considerable scientific impact in VLS‐related disciplines (Table 1).

**Table 1:** The top 10 countries that contributed publications on vulvar lichen sclerosus.

Rank	Country	Total publications	Total citations	Average citations	H‐index
1	USA	485	6196	21,34	50
2	UK	221	4030	34.17	45
3	Italy	208	3461	25.5	38
4	Germany	115	3168	35.26	36
5	China	93	495	8.51	13
6	Australia	89	1292	21.75	25
7	Netherlands	74	1,716	35.18	28
8	India	66	671	14.21	15
9	Spain	59	1143	22.66	21
10	Canada	58	699	15.48	17

Abbreviations: UK, United Kingdom; USA, United States.

**Figure 3:**
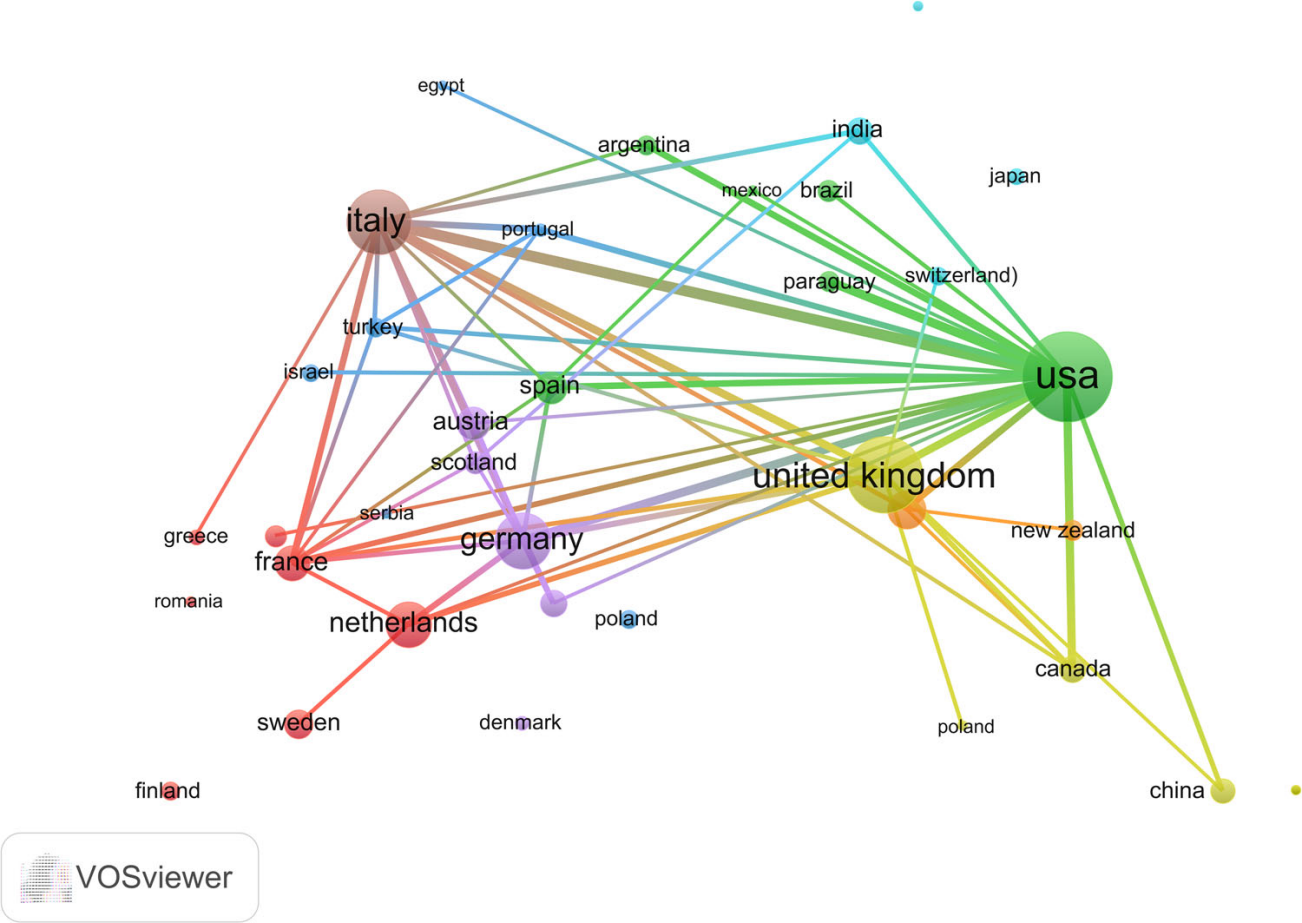
Cooperation network of countries.

The findings of the network correlation analysis provide additional evidence supporting the prominent roles of the USA, UK, Italy, Germany, and Netherlands in the field of VLS‐related research. Particularly noteworthy are the substantial collaborations between the USA and Italy, as well as between the USA and Germany, as illustrated in Figure 3. Moreover, the presence of strong connections between other countries suggests opportunities for enhancing international cooperation in this area (Figure 3).

### 3.2 Contributions of Institutions

The top 10 institutions were identified based on their performance in publication count, total citations, average citations, and H‐index (Table 2). Among these institutions, the University of London distinguished itself with 78 publications and 2160 citations. Following closely were University College London with 48 publications, the University of California System with 45 publications, and the University of Oxford with 44 publications. The University of London exhibited the highest citation count and H‐index among the top institutions.

**Table 2:** The top 10 Institution that contributed publications on Vulvar Lichen Sclerosus.

Rank	Institution	Country	Total publications	Total citations	Average citations	H‐index
1	University of London	UK	78	2160	36.58	28
2	University college London	UK	48	1160	27.06	18
3	University of Clifornia System	USA	45	795	19,56	16
4	University of Oxford	UK	44	1377	62.41	27
5	Harvard University	USA	42	1187	36.74	26
6	University of Ferrara	Italy	34	319	17.59	15
7	University of Texas System	USA	33	541	17.52	14
8	University of Florence	Italy	32	873	38.53	16
9	Assistance Publique Hopitaux Paris Aphp	France	29	1145	42.28	16
10	Harvard Medical School	USA	29	769	34.21	21

Abbreviations: UK, United Kingdom; USA, United States.

**Figure 4:**
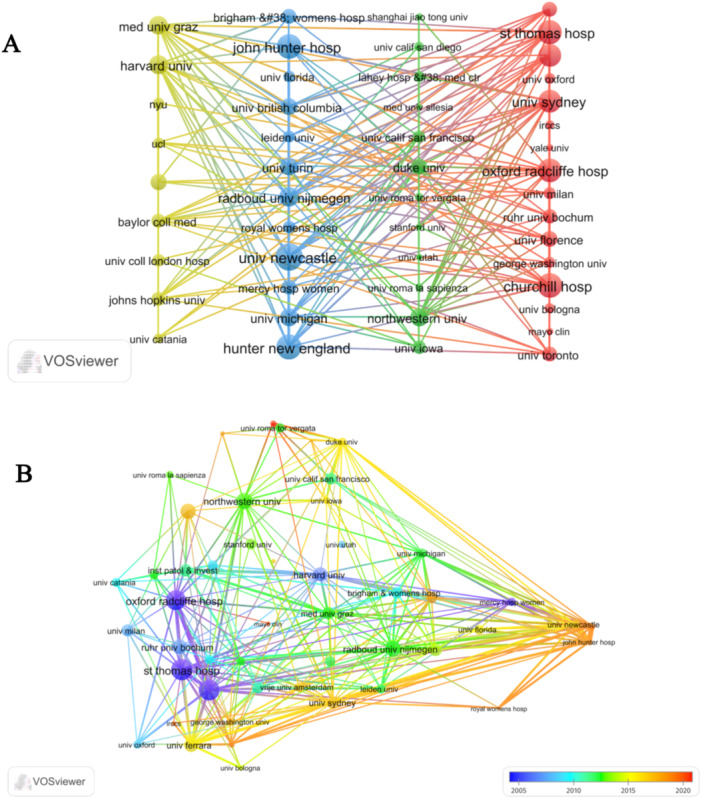
Network map of interinstitutional cooperation (A) network visualization; (B) overlay visualization.

The results of the cooperation network analysis revealed that Oxford Radcliffe Hospital, St. Thomas Hospital, University of Sydney, John Hunter Hospital, and University of Newcastle exhibited a significantly greater level of influence in the field of VLS‐related research in comparison to other institutions. Additionally, the analysis emphasized that interinstitutional collaborations predominantly occurred within national borders, leading to limited instances of international cooperation (Figure 4A). In recent years, network analysis has shown a growing focus on the field of VLS by the University of Newcastle and John Hunter Hospital, as evidenced by the gradual increase in the number of publications (Figure 4B).

### 3.3 Contributions of Authors

**Figure 5:**
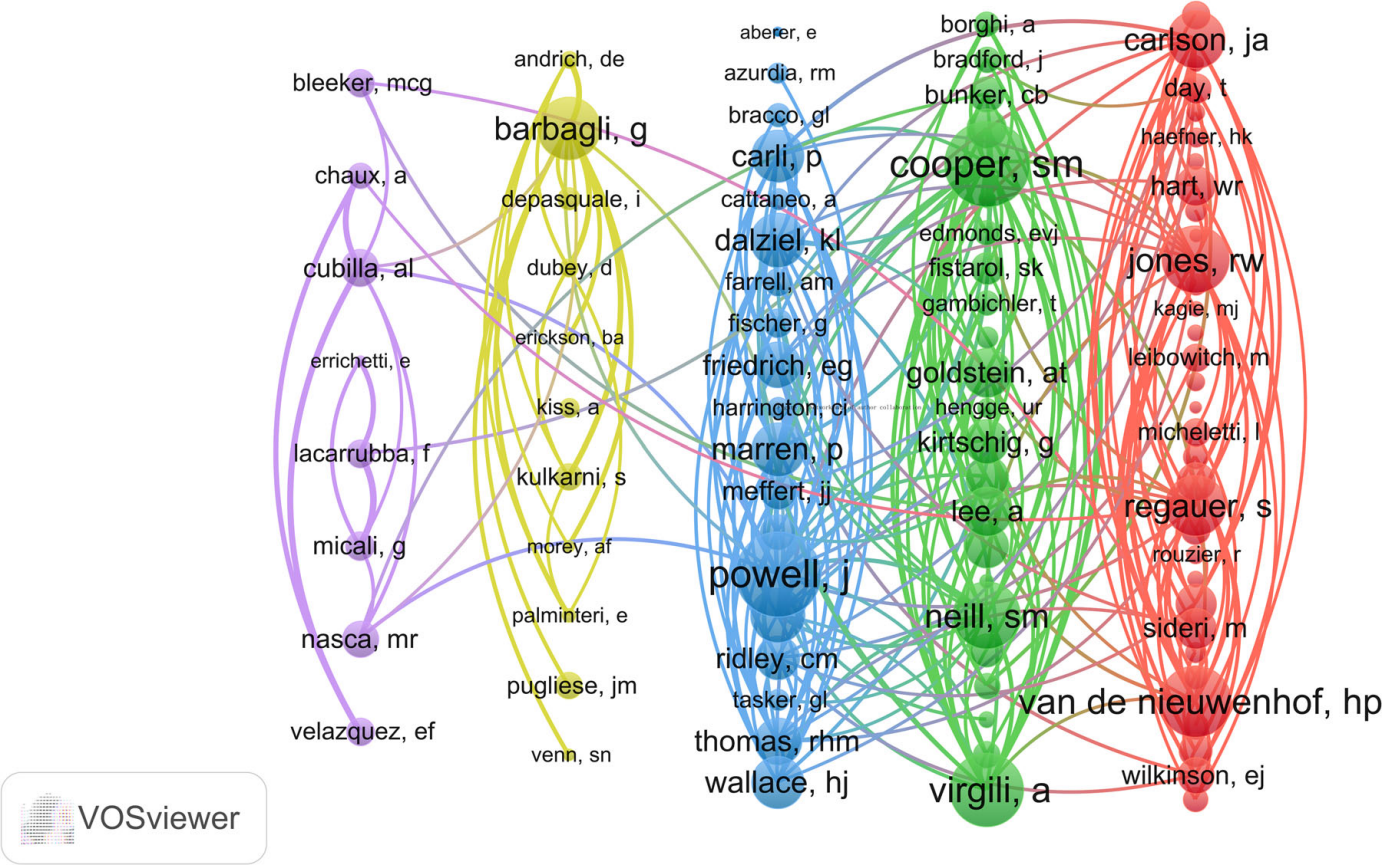
Network map of author collaboration.

Based on an analysis of publication count, total citations, average citations, and H‐index, the top 10 authors were identified and recognized for their exceptional performance, as shown in Table 3. Among these authors, Wojnarowska, F (39 articles), Corazza, M (33 articles), Borghi, A (30 articles), Scurry, J (24 articles), and Barbagli, G (21 articles) were found to be the top five most productive authors. Of particular note, Wojnarowska, F consistently ranked first in terms of publications, total citations, and H‐index, while Lazzeri, Massimo consistently ranked first on average citations, highlighting their significant impact in the field. Furthermore, the current study analyzed the collaboration among these prolific authors, as illustrated in Figure 5. The cooperation map emphasizes the strong collaborative ties among international research teams, demonstrating their active engagement in academic endeavors.

**Table 3:** The top 10 authors that contributed publications on vulvar lichen sclerosus.

Rank	Authors	Total publications	Total citations	Average citations	H‐index
1	Wojnarowska, F	39	1238	66.67	25
2	Corazza, Monica	33	308	17.79	15
3	Borghi, Alessandro	30	245	16.5	14
4	Scurry, James	24	500	29	14
5	Barbagli, G.	21	965	68.14	17
6	Bunker, Chris B.	19	343	23.37	9
7	Fischer, Gayle	19	399	29.79	10
8	Minghetti, Sara	18	168	19.33	12
9	De Hullu, J. A.	18	512	41.22	12
10	Lazzeri, Massimo	17	844	70.29	13

### 3.4 Contributions of Journals

**Figure 6:**
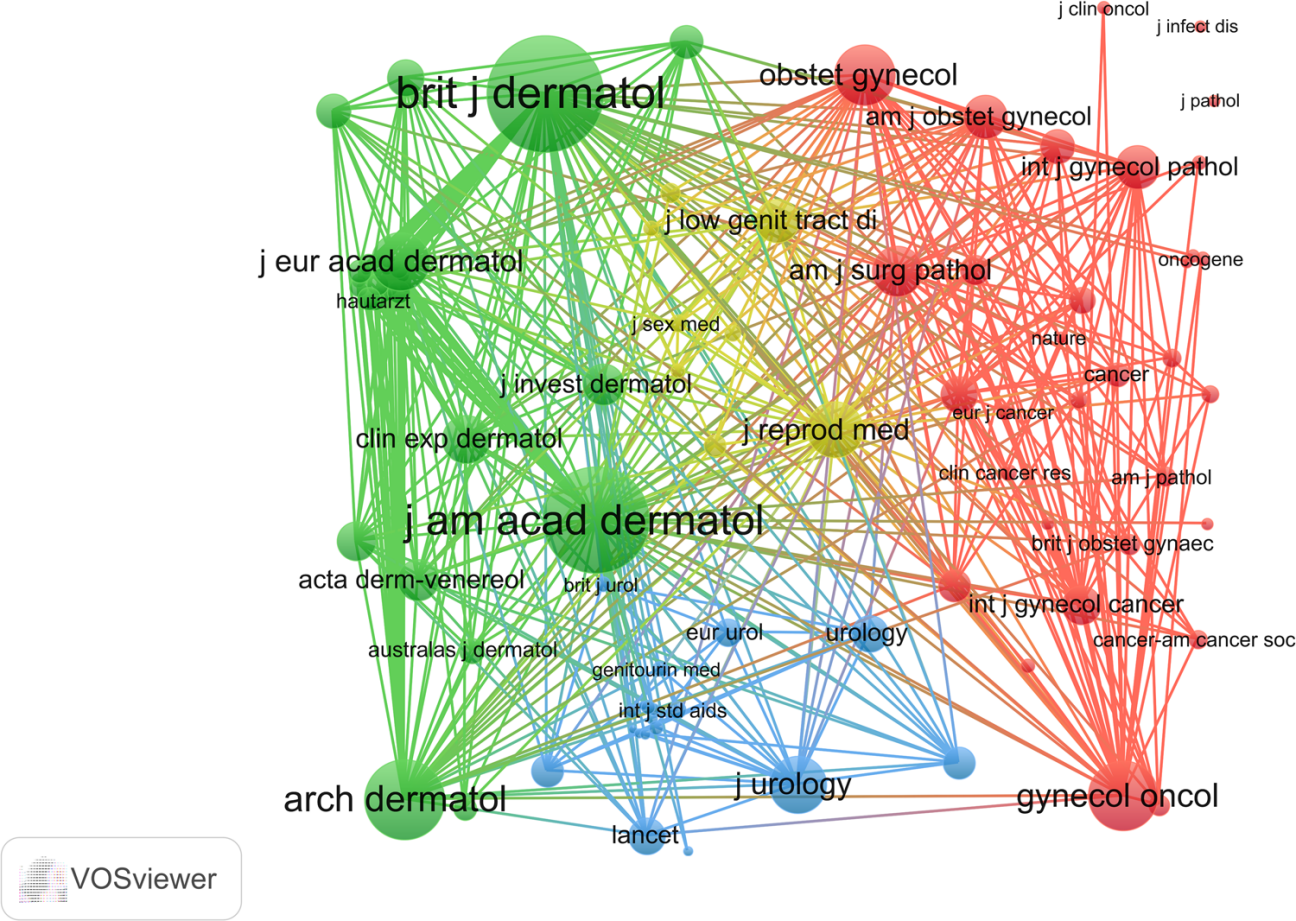
Visualization map of co‐cited journals.

Within the field of VLS research, this study has identified the 10 most prominent and impactful journals, with a notable concentration of these top journals originating from the USA (Tables 4, 5). The Journal of Lower Genital Tract Disease was found to be the most active publication over the last three decades. Additionally, the Journal of British Journal of Dermatology, Journal of the American Academy of Dermatology, Journal of Urology, and Journal of the European Academy of Dermatology and Venereology achieved a Q1 ranking in the JCR quartile, while the Journal of Lower Genital Tract Disease was classified as Q2. The remaining journals were classified in the third and fourth quartiles. These results suggest that the overall standard of research in the field is satisfactory. Particularly noteworthy is the high Co‐Citations and total link strength received by the British Journal of Dermatology and Journal of the American Academy of Dermatology, indicating the esteem in which articles published in these journals are held, despite their classification in the first quartile. Network analysis further confirmed the significant influence of the British Journal of Dermatology, Journal of the American Academy of Dermatology, Archives of Dermatology, and Gynecologic Oncology in VLS research (Figure 6).

**Table 4:** The top 10 journals that contributed publications on vulvar lichen sclerosus.

Rank	Journal	Articles	Country	JCR	IF
1	Journal of Lower Genital Tract Disease	74	USA	Q2	3.7
2	British Journal of Dermatology	51	UK	Q1	10.3
3	Journal of the American Academy of Dermatology	42	USA	Q1	13.8
4	Journal of Urology	42	USA	Q1	6.6
5	American Journal of Dermatopathology	39	USA	Q4	1.1
6	Journal of Reproductive Medicine	37	USA	Q4	0.2
7	International Journal of Gynecological Pathology	36	USA	Q3	2.4
8	Journal of the European Academy of Dermatology and Venereology	36	Netherlands	Q1	9.2
9	Journal of Cutaneous Pathology	35	Denmark	Q3	1.7
10	Urology	35	USA	Q3	2.1

Abbreviations: UK, United Kingdom; USA, United States.

**Table 5:** The top 10 influential journals in the field of Vulvar Lichen Sclerosus.

Rank	Co‐cited journal	Country	Co‐citations	Total link strength	JCR	IF
1	British Journal of Dermatology	UK	2697	96423	Q1	10.3
2	Journal of the American Academy of Dermatology	USA	2356	83684	Q1	13.8
3	Journal of Urology	USA	1756	34941	Q1	6.6
4	Archives of Dermatology	USA	1554	56451	—	—
5	Gynecologic Oncology	USA	1102	46857	Q1	4.7
6	Journal of Reproductive Medicine	USA	1097	34377	Q4	0.2
7	Obstetrics and Gynecology	USA	979	38081	Q1	7.2
8	Journal of the European Academy of Dermatology and Venereology	UK	874	35594	Q1	9.2
9	American Journal of Surgical Pathology	USA	771	28728	Q1	5.6
10	Clinical and Experimental Dermatology	UK	693	26950	Q1	4.1

Abbreviations: UK, United Kingdom; USA, United States.

### 3.5 Co‐Cited Reference and Co‐Cited Articles Cluster Analysis

**Figure 7:**
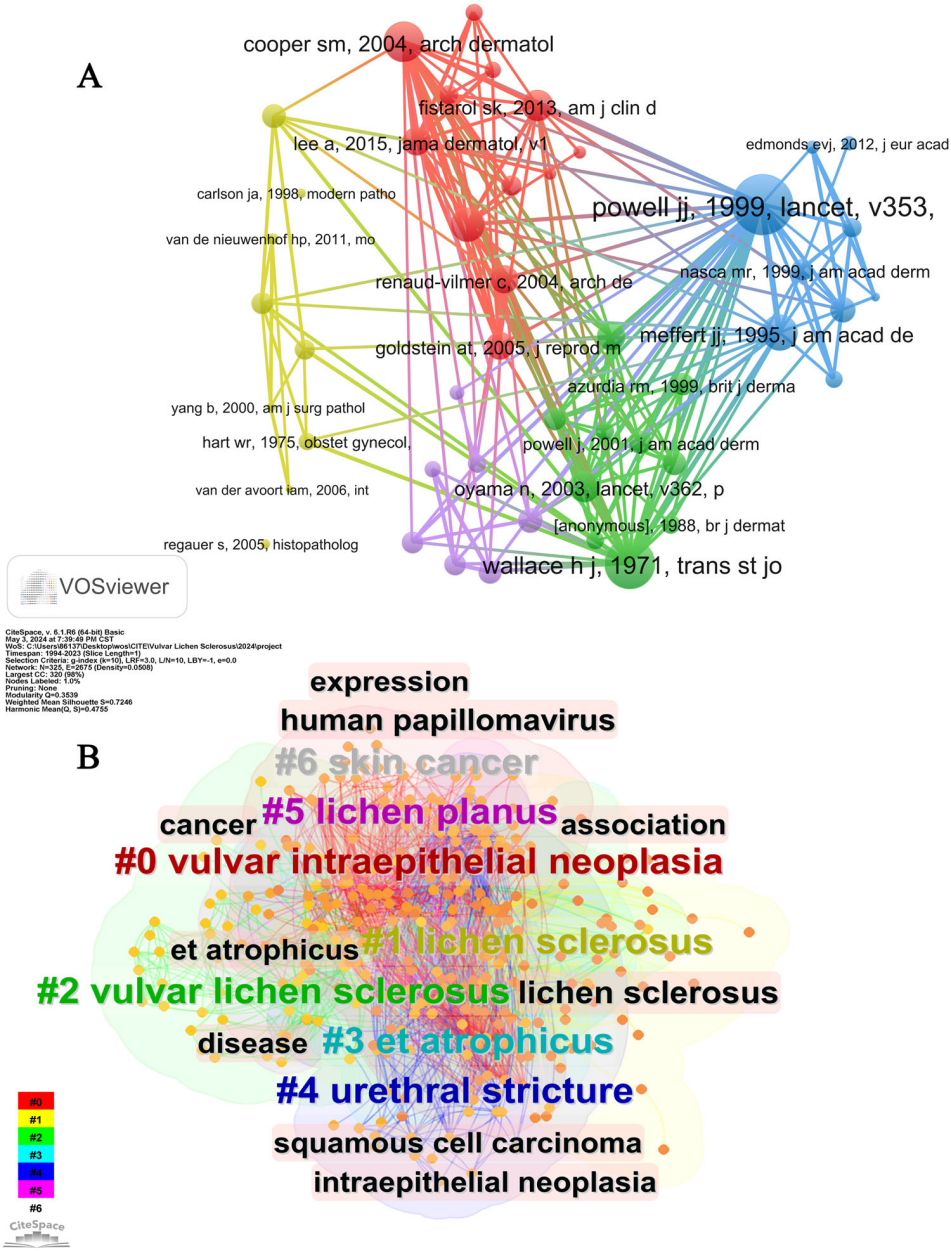
Visualization map of co‐cited references (A) and keywords clustering (B).

Literary analysis is essential for identifying research frontiers and establishing foundational knowledge in academic research. Co‐citation analysis, a commonly utilized method for determining relationships between scholarly articles through shared citations, was employed in this study using Citespace software to generate co‐citation correlation and cluster network maps. The analysis encompassed 25,847 cited references, with particular emphasis on identifying the most frequently cited sources (Figure 7A). The cluster map analysis (*Q* = 0.3539, *S* = 0.7246) identified ten prominent themes in VLS research spanning a 30‐year period. These themes include “Vulvar intraepithelial neoplasia,” “Lichen sclerosus,” “Vulvar lichen sclerosus,” “Et atrophicus,” “Urethral stricture,” “Lichen planus,” and “Skin cancer” as illustrated in Figure 7B. The cluster map was considered satisfactory in quality as indicated by *Q* > 0.3 and *S* > 0.5, thereby enhancing the credibility of the study.

The present research has identified the 10 most frequently cited articles, predominantly published in esteemed journals such as The Lancet, Journal of the American Academy of Dermatology, American Journal of Clinical Dermatology, JAMA Dermatology, and British Journal of Dermatology (Table 6). Notably, the article entitled “Lichen sclerosus,” authored by Powell and Wojnarowska [27], received the most citations (*n* = 225) and was published in 1999. The remaining articles, published between 1998 and 2015, had citation counts ranging from 80 to 154. Among the top 6 of the 10 studies, the primary focus was on investigating the efficacy and safety of TCS in the treatment of LS, with these medications being considered as the main treatment for VLS [27, 28, 29, 30, 31, 32]. Goldstein et al.'s study, which included 1675 patients, identified 28 cases (1.7%) of confirmed VLS through biopsy. The study emphasized the importance of maintaining a high suspicion index for diagnosis, as a significant portion of patients may be asymptomatic [7]. Three additional articles further explored the etiology and potential mechanisms of VLS malignancy. One study presented findings supporting a distinct humoral immune response to extracellular matrix protein 1 in LS [33]. Alterations in the p53 gene appeared to be associated with the progression of simple vulvar intraepithelial neoplasia (VIN) and potentially contributed to the carcinogenic process [34]. The third article conducted a literature review on male LS from 1950 to 2006, suggesting that biopsy of all suspected LS patients is necessary to exclude SCC [35]. Ultimately, uncertainties persist regarding the most effective treatment regimen for VLS and its potential for malignancy, necessitating additional research.

**Table 6:** Top 10 co‐cited references in the fields of vulvar lichen sclerosus.

Rank	Author	Citations	Title	Journal	Year
1	Powell J.J. [27]	225	Lichen sclerosus	Lancet	1999
2	Meffert J.J. [28]	154	Lichen‐sclerosus	Journal of the American Academy of Dermatology	1995
3	Fistarol S.K. [29]	132	Diagnosis and Treatment of Lichen Sclerosus An Update	American Journal of Clinical Dermatology	2013
4	Lee A. [30]	112	Long‐term Management of Adult Vulvar Lichen Sclerosus A Prospective Cohort Study of 507 Women	Jama Dermatology	2015
5	Neill S.M. [31]	109	British Association of Dermatologists' guidelines for the management of lichen sclerosus 2010	British Journal of Dermatology	2010
6	Cooper S.M. [32]	106	Does treatment of vulvar lichen sclerosus influence its prognosis?	Archives of Dermatology	2004
7	Oyama N. [33]	99	Autoantibodies to extracellular matrix protein 1 in lichen sclerosus	Lancet	2003
8	Carlson J.A. [34]	94	Vulvar lichen sclerosus and squamous cell carcinoma: A cohort, case control, and investigational study with historical perspective; Implications for chronic inflammation and sclerosis in the development of neoplasia	Human Pathology	1998
9	Pugliese J.M. [35]	93	Lichen sclerosus: Review of the literature and current recommendations for management	Journal of Urology	2007
10	Goldstein A.T. [7]	80	Prevalence of vulvar lichen sclerosus in a general gynecology practice	Journal of Reproductive Medicine	2005

### 3.6 The Mechanism of VLS

**Figure 8:**
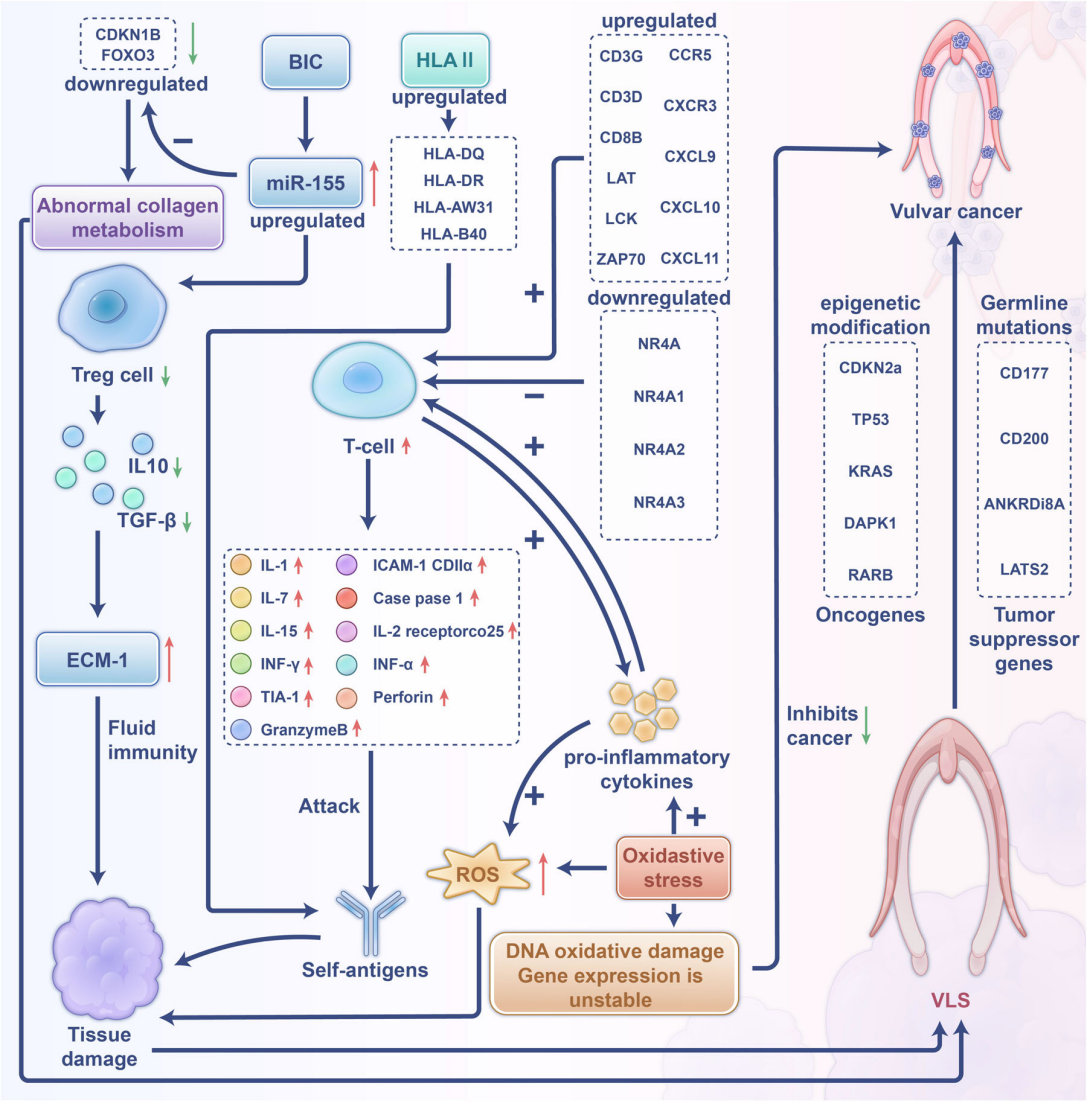
Mechanism diagram of vulvar lichen sclerosus.

The pathogenesis of VLS remains incompletely elucidated. A review of the literature identifies several pathways implicated in the onset of VLS (Figure 8). First, patients with VLS exhibit genetic susceptibility [36, 37]. Upon exposure to specific triggering factors [17, 38], aberrant gene expression may modulate T cells and other immunological components [39, 40], culminating in cellular and humoral immune dysfunction. This dysfunction is characterized by elevated levels of pro‐inflammatory cytokines and altered self‐antigen presentation. Consequently, these cytokines target self‐antigens in the external genital tissues, resulting in tissue damage and the manifestation of disease. Second, oxidative stress has been implicated in a cascade of detrimental effects, including oxidative damage to DNA and proteins, which subsequently results in tissue damage, disease, unstable gene expression, and the downregulation of tumor suppressor genes. This series of events may increase the risk of VLS progressing to vulvar cancer. Additionally, oxidative stress may play a crucial role in the initiation, maintenance, and progression of chronic inflammation in VLS, contributing to the autoimmune processes associated with the condition and leading to tissue sclerosis and scarring [41]. Finally, individuals with a genetic predisposition to VLS may exhibit epigenetic modifications or germline mutations, potentially contributing to the pathogenesis of vulvar cancer [42].

### 3.7 Future Trends

**Figure 9:**
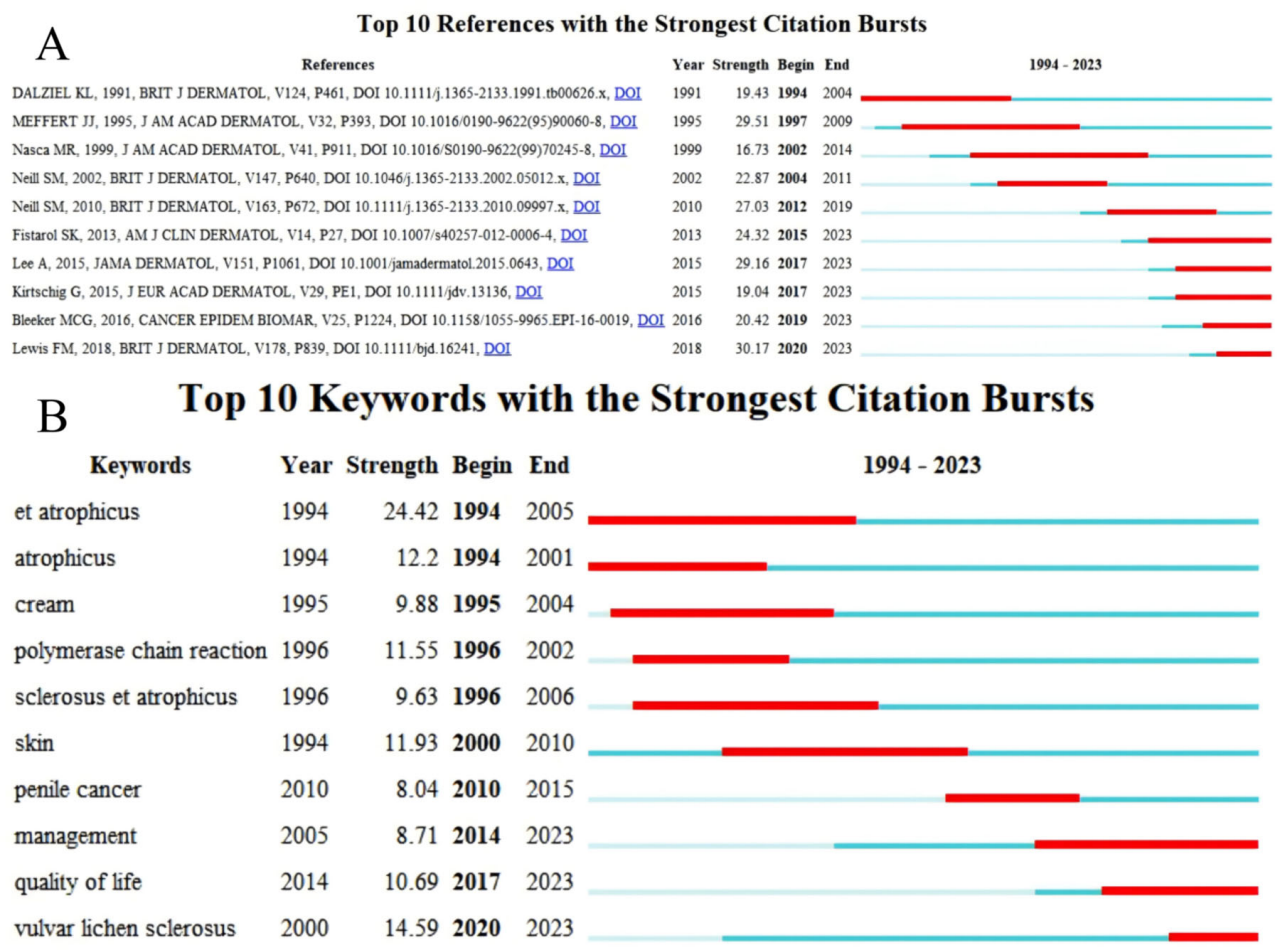
Top 10 references and keywords with the strongest citation bursts.

In the current research, the Citespace software was utilized to identify the top 10 references with significant citation bursts within the last three decades. The presence of a substantial citation burst indicates a notable level of interest from the scientific community and sheds light on emerging trends or topics within the field of VLS. These selected papers demonstrated burst strengths ranging from 16.73 to 30.17, with burst durations spanning from 4 to 13 years (Figure 9A). Notably, the top five articles in terms of burst strengths predominantly focused on the prevention and treatment of TCS in VLS. The top‐ranked articles included literature reviews and guidelines that provided evidence supporting the therapeutic efficacy of TCS in the treatment of VLS [12, 28]. One study emphasized the effectiveness of individualized preventive TCS regimens in managing VLS [30]. Another article, ranking fourth, demonstrated sustained impact over a 13‐year period (1997–2009) and further supported the efficacy of TCS [31]. The fifth‐ranked article, with a high burst strength and publication span from 2017 to 2023, highlighted the continued importance of potent and ultrapotent TCS in the therapeutic approach to VLS [29]. The aforementioned studies have demonstrated that the treatment of VLS with TCS has garnered significant clinical interest.

**Figure 10:**
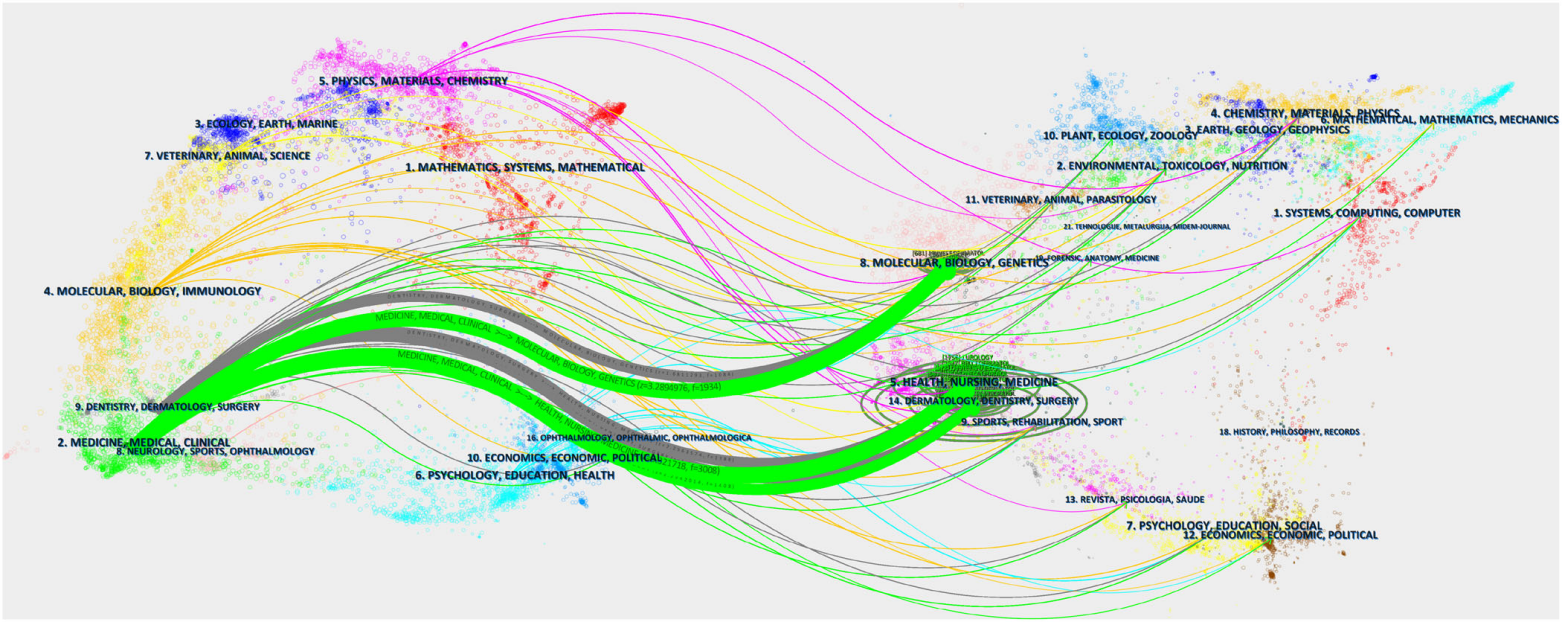
Dual‐map overlays.

Furthermore, the current study has identified the top 10 keywords exhibiting the most pronounced citation bursts, emphasizing atrophicus and VLS as persistent areas of focus (Figure 9B). CiteSpace has developed a dual‐map overlay utilizing matrices of citing and cited journals. In this visualization, the left side represents the citing journals or topics, while the right side depicts the cited journals or topics. Figure 10 in this study indicates that the majority of publications originated from the field on the left (“Medicine, Medical and Clinical”), which was predominantly influenced by the field on the right, specifically “Molecular, Biology, Genetics” (*z* = 3.2895, *f* = 1934), “Health, Nursing, Medicine” (*z* = 6.3217, *f* = 3008), and “Dermatology, Dentistry, Surgery” (*z* = 2.2942, *f* = 1408). Additionally, the left domain, “Dermatology, Dentistry, Surgery,” was mainly influenced by the right domain, comprising “Molecular, Biology, Genetics” (*z* = 1.6811, *f* = 1084) and “Health, Nursing, Medicine” (*z* = 2.2563, *f* = 1388). Here, f denotes the frequency of citation, and z represents the standardized values of f (Figure 10).

Integrating the average appearing year of keywords in VOSviewer with the Citation Explosion time zone map in CiteSpace reveals a transformative shift in research focus. The research focus has gradually shifted from studying previous pathological changes such as “epidermal atrophy” to emphasizing “long‐term management of treatment” and improving patients “quality of life” (QOL), as well as studying the unified naming of this disease “vulvar lichen sclerosus” and the risk factors for its onset. In summary, it is expected that various institutions will allocate more resources for the prevention and long‐term standardized management of VLS.

## 4 Discussion

LS is a persistent dermatologic disorder distinguished by significant inflammation, thinning of the epithelium, and specific alterations in the dermis. VLS may lead to depigmentation, atrophy, and fissures on the anogenital skin, often accompanied by severe itching or discomfort. Additionally, VLS can lead to functional impairment, disfigurement, and diminished QOL. Thus, the prevention and management of VLS progression are crucial. Early intervention for VLS is recommended whenever possible [43]. TCS represent the primary treatment modality for VLS. A prospective cohort study involving 507 adult patients demonstrated that sustained adherence to TCS therapy was correlated with reduced incidences of adhesions, scarring, and VIN or SCC [30]. The recommended clinical protocol involves nightly application of the medication for a duration of 12 weeks, with the objective of achieving disease remission. The tapering regimen outlined in the 2018 British Association of Dermatologists guidelines involves a gradual reduction in the frequency of application over a period of 12 weeks [44]. Despite medical treatment, atrophic anatomical changes such as resorption of the labia minora and scarring often remain. Surgical intervention and physical therapy may be effective in improving disabling adhesions or scarring.

The present study encompassed a total of 1698 articles pertaining to VLS. Results indicated a notable rise in VLS‐related publications post‐2018, predominantly emanating from the USA and UK. Germany and Netherlands emerged as frontrunners on average citations, suggesting superior quality of research output in this domain. Close international collaboration was observed in the VLS field, notably among the USA, UK, Italy, Germany, and Netherlands. Accordingly, it is crucial to continuously enhance international cooperation in the VLS field in the future.

By evaluating institutions and authors, significant insights were gained regarding their contributions and impact in the field of VLS. The University of London excelled in the number of VLS‐related articles, H‐index, and total citations, surpassing other institutions. Additionally, the University of Oxford and Assistance Publique Hopitaux Paris Aph ranked highest for average citations, suggesting their production of high‐quality research. In the realm of author analysis, scholars affiliated with the University of Oxford have played a significant role, with Wojnarowska, F being particularly influential, leading in total publications, total citations, and H‐index. It is noteworthy, however, that Lazzeri, Massimo and Barbagli, G rank among the top two in terms of average citations. The network map of author collaborations reveals a pattern of frequent domestic collaboration and relatively close international cooperation within this academic domain.

In the course of journal analysis, a variety of IFs ranging from 0.2 to 13.8 were noted among the top 10 journals. It is noteworthy that the majority of these journals cater to the specialized field of VLS, targeting a niche audience with restricted outreach. Therefore, it is imperative to recognize that IF alone should not be the exclusive determinant of research excellence. By employing the JCR, this study identified four journals ranked in the first quartile based on publication volume and eight journals ranked in the first quartile based on citations. This indicates that a significant proportion of articles related to VLS exhibited a high level of research quality, particularly those published in the British Journal of Dermatology, Journal of the American Academy of Dermatology, and Journal of Urology, which garnered substantial citation counts.

The present study investigated the prevailing research themes and patterns in the domain of VLS. Through citation burst analysis, it was observed that a significant portion of the top 10 studies concentrated on assessing the efficacy and safety of TCS as a primary therapeutic intervention for LS within the context of VLS. This observation is consistent with the findings obtained from co‐citation analysis. Consequently, the current research trends in the field of VLS primarily revolve around the evaluation of the effectiveness and safety of TCS in managing LS, along with the identification of optimal approaches for its utilization. Additional treatments that have shown potential benefits in limited patient populations include oral acitretin [45], oral methotrexate [46], light‐based therapies such as ultraviolet A1 phototherapy [47], photodynamic therapy [48], and laser therapy [49], as well as the use of silk underpants [50], topical tretinoin [51], oral cyclosporine [52], oral adalimumab [53], and treatment with platelet‐rich plasma [54]. However, further research is needed to substantiate the efficacy of these treatments.

Three of the top 10 articles examined the etiology and potential mechanisms of VLS malignancy. Adult‐acquired VLS is an important risk factor for vulvar SCC [10]. Concurrent VLS is frequently observed in patients with vulvar SCC [55]. The risk of vulvar SCC in children with VLS remains uncertain, although it is believed to be unlikely [56]. Incidences of SCC in extragenital LS sites are infrequent [57]. Males with penile LS may have an increased risk of penile SCC. Current research focuses on identifying LS cases with a higher likelihood of progressing to SCC [14]. The risk of vulvar SCC may be increased in VLS patients. Effective management of this condition may be crucial in mitigating the risk of neoplastic development, with TCS emerging as the primary clinical treatment modality.

The primary treatment for VLS continues to be ultrapotent TCS, accompanied by long‐term maintenance therapy. However, from a clinical perspective, there is a prevalent phenomenon of steroid phobia among patients [58]. Recent research has increasingly concentrated on the impact of VLS on QOL [59], and various scales have been developed to assess the burden of vulvar disease [60]. Given the current ambiguity surrounding the exact pathogenesis of VLS [61], a variety of treatment modalities are employed. Although numerous novel therapies are being investigated to alleviate symptoms and enhance QOL, future research is anticipated to concentrate on elucidating the disease's etiology and underlying mechanisms. This focus aims to achieve a more comprehensive understanding of VLS pathogenesis and to identify potential therapeutic targets.

The current investigation possesses various noteworthy limitations. Primarily, the data for the study were acquired through software tools, potentially leading to bias [62]. Furthermore, this study exclusively utilized the WOS database to search for relevant literature, which may not encompass all published works related to VLS. Finally, the analysis was restricted to English‐language literature, excluding non‐English publications, which could potentially influence the research outcomes.

## 5 Conclusions

Since 2018, VLS publications in SCI have surged, with the USA and UK emerging as leading contributors. Research has moved from examining past pathological changes like “epidermal atrophy” to focusing on “long‐term treatment management,” improving “quality of life,” standardizing the disease name “vulvar lichen sclerosus,” and identifying risk factors. Institutions are expected to allocate more resources for VLS prevention and long‐term management. The exact mechanism of VLS remains unclear, but it is linked to cytokine immune regulation, oxidative stress, and potential epigenetic changes or genetic mutations in susceptible individuals. It could also develop into vulvar squamous cell carcinoma.

## Author Contributions

**Fan‐Rong He:** data curation, software. writing – original draft. **Yan‐Rui Lin:** formal analysis, validation. **Yu‐Hua Wen:** methodology, visualization. **Jin‐He Deng:** conceptualization; methodology, resources, supervision, writing – review and editing.

## Conflicts of Interest

The authors declare no conflicts of interest.

## Transparency Statement

The lead author Jin‐He Deng affirms that this manuscript is an honest, accurate, and transparent account of the study being reported; that no important aspects of the study have been omitted; and that any discrepancies from the study as planned (and, if relevant, registered) have been explained.

## Supporting information

Table S1.

## Data Availability

The authors confirm that the data supporting the findings of this study are available within the article and its supporting materials.

## References

[1] D. A. De Luca , C. Papara , A. Vorobyev , et al., “Lichen Sclerosus: The 2023 Update,” Frontiers in Medicine 10 (2023): 1106318, 10.3389/fmed.2023.1106318. 36873861 PMC9978401

[2] F. Tasker , L. Kirby , D. J. C. Grindlay , F. Lewis , and R. C. Simpson , “Laser Therapy for Genital Lichen Sclerosus: A Systematic Review of the Current Evidence Base,” Skin Health and Disease 1, no. 3 (2021): e52, 10.1002/ski2.52. 35663131 PMC9060003

[3] P. Campolmi , G. Cannarozzo , L. Bennardo , A. Clementi , M. Sannino , and S. P. Nisticò , “Fractional Micro‐Ablative CO2 Laser as Therapy in Penile Lichen Sclerosus,” Journal of Lasers in Medical Sciences 12 (2021): e61, 10.34172/jlms.2021.61. 35155146 PMC8837861

[4] H. J. Wallace , “Lichen Sclerosus et Atrophicus,” Transactions of the St. John's Hospital Dermatological Society 57, no. 1 (1971): 9–30. 5570266

[5] R. W. Jones , J. Scurry , S. Neill , and A. B. MacLean , “Guidelines for the Follow‐Up of Women With Vulvar Lichen Sclerosus in Specialist Clinics,” American Journal of Obstetrics and Gynecology 198, no. 5 (2008): 496.e1–496.e3, 10.1016/j.ajog.2007.05.031. 17905173

[6] G. O. Fischer , “The Commonest Causes of Symptomatic Vulvar Disease: A Dermatologist's Perspective,” Australasian Journal of Dermatology 37, no. 1 (1996): 12–18, 10.1111/j.1440-0960. 8936065

[7] A. T. Goldstein , S. C. Marinoff , K. Christopher , and M. Srodon , “Prevalence of Vulvar Lichen Sclerosus in a General Gynecology Practice,” Journal of Reproductive Medicine 50, no. 7 (2005): 477–480. 16130842

[8] A. Leibovitz , V. Kaplun , N. Saposhnicov , and B. Habot , “Vulvovaginal Examinations in Elderly Nursing Home Women Residents,” Archives of Gerontology and Geriatrics 31, no. 1 (2000): 1–4, 10.1016/s0167-4943(00)00059-5. 10989157

[9] A. Lee and G. Fischer , “Diagnosis and Treatment of Vulvar Lichen Sclerosus: An Update for Dermatologists,” American Journal of Clinical Dermatology 19, no. 5 (2018): 695–706, 10.1007/s40257-018-0364-7. 29987650

[10] M. C. G. Bleeker , P. J. Visser , L. I. H. Overbeek , M. van Beurden , and J. Berkhof , “Lichen Sclerosus: Incidence and Risk of Vulvar Squamous Cell Carcinoma,” Cancer Epidemiology, Biomarkers & Prevention 25, no. 8 (2016): 1224–1230, 10.1158/1055-9965.EPI-16-0019. 27257093

[11] P. Halonen , M. Jakobsson , O. Heikinheimo , M. Gissler , and E. Pukkala , “Incidence of Lichen Sclerosus and Subsequent Causes of Death: A Nationwide Finnish Register Study,” BJOG: An International Journal of Obstetrics & Gynaecology 127, no. 7 (2020): 814–819, 10.1111/1471-0528.16175. 32065721

[12] F. M. Lewis , F. M. Tatnall , S. S. Velangi , et al., “British Association of Dermatologists Guidelines for the Management of Lichen Sclerosus, 2018,” British Journal of Dermatology 178, no. 4 (2018): 839–853, 10.1111/bjd.16241. 29313888

[13] P. Vieira‐Baptista , F. R. Pérez‐López , M. T. López‐Baena , C. K. Stockdale , M. Preti , and J. Bornstein , “Risk of Development of Vulvar Cancer in Women With Lichen Sclerosus or Lichen Planus: A Systematic Review,” Journal of Lower Genital Tract Disease 26, no. 3 (2022): 250–257, 10.1097/LGT.0000000000000673. 35285455

[14] M. R. Raspollini , G. Asirelli , D. Moncini , and G. L. Taddei , “A Comparative Analysis of Lichen Sclerosus of the Vulva and Lichen Sclerosus That Evolves to Vulvar Squamous Cell Carcinoma,” American Journal of Obstetrics and Gynecology 197, no. 6 (2007): 592.e1–592.e5, 10.1016/j.ajog.2007.04.003. 17714682

[15] J. Powell and F. Wojnarowska , “Childhood Vulvar Lichen Sclerosus: An Increasingly Common Problem,” Journal of the American Academy of Dermatology 44, no. 5 (2001): 803–806, 10.1067/mjd.2001.113474. 11312428

[16] R. H. M. Thomas , C. M. Ridley , D. H. McGibbon , and M. M. Black , “Lichen Sclerosus et Atrophicus and Autoimmunity‐‐A Study of 350 Women,” British Journal of Dermatology 118, no. 1 (1988): 41–46, 10.1111/j.1365-2133.1988.tb01748.x. 3342175

[17] K. Eisendle , T. Grabner , H. Kutzner , and B. Zelger , “Possible Role of Borrelia Burgdorferi Sensu Lato Infection in Lichen Sclerosus,” Archives of Dermatology 144, no. 5 (2008): 591–598, 10.1001/archderm.144.5.591. 18490585

[18] Y. Soini , P. Pääkkö , K. Vähäkangas , S. Vuopala , and V. P. Lehto , “Expression of p53 and Proliferating Cell Nuclear Antigen in Lichen Sclerosus et Atrophicus With Different Histological Features,” International Journal of Gynecological Pathology 13, no. 3 (1994): 199–204, 10.1097/00004347-199407000-00002. 7928051

[19] A. R. Günthert , K. Duclos , B. G. Jahns , et al., “Clinical Scoring System for Vulvar Lichen Sclerosus,” The Journal of Sexual Medicine 9, no. 9 (2012): 2342–2350, 10.1111/j.1743-6109.2012.02814.x. 22759453

[20] S. Naswa and Y. Marfatia , “Physician‐Administered Clinical Score of Vulvar Lichen Sclerosus: A Study of 36 Cases,” Indian Journal of Sexually Transmitted Diseases and AIDS 36, no. 2 (2015): 174–177, 10.4103/0253-7184.167169. 26692611 PMC4660559

[21] C. Chen , “Citespace II: Detecting and Visualizing Emerging Trends and Transient Patterns in Scientific Literature,” Journal of the American Society for Information Science and Technology 57 (2006): 359–377, 10.1002/asi.20317.

[22] N. J. Van Eck and L. Waltman , “Software Survey: VoSviewer, a Computer Program for Bibliometric Mapping,” Scientometrics 84, no. 2 (2010): 523–538, 10.1007/s11192-009-0146-3. 20585380 PMC2883932

[23] H. Yan , S. Zhang , W. Sun , et al., “A Bibliometric and Visual Analysis of the Research Status and Hotspots of Seborrheic Dermatitis Based on Web of Science,” Skin Research and Technology 30, no. 9 (2024): e70048, 10.1111/srt.70048. 39252564 PMC11386261

[24] Y. Yang , X. Zheng , H. Lv , et al., “A Bibliometrics Study on the Status Quo and Hot Topics of Pathogenesis of Psoriasis Based on Web of Science,” Skin Research and Technology 30, no. 1 (2024): e13538, 10.1111/srt.13538. 38174774 PMC10765367

[25] W. Zuo , Z. Yue , S. Xu , et al., “Emerging Trends and Research Hotspots in the Relationship Between Mast Cells and Atopic Dermatitis Based on the Literature From 2001 to 2024: A Bibliometric and Visualized Analysis,” Skin Research and Technology 30, no. 9 (2024): e70053, 10.1111/srt.70053. 39234634 PMC11375331

[26] J. Chen , C. Luo , P. Ju , et al., “A Bibliometric Analysis and Visualization of Acupuncture and Moxibustion Therapy for Herpes Zoster and Postherpetic Neuralgia,” Skin Research and Technology 30, no. 6 (2024): e13815, 10.1111/srt.13815. 38924142 PMC11197023

[27] J. Powell and F. Wojnarowska , “Lichen Sclerosus,” The Lancet 353, no. 9166 (1999): 1777–1783, 10.1016/s0140-6736(98)08228-2. 10348006

[28] J. J. Meffert , B. M. Davis , and R. E. Grimwood , “Lichen Sclerosus,” Journal of the American Academy of Dermatology 32, no. 3 (1995): 393–416, 10.1016/0190-9622(95)90060-8. 7868709

[29] S. K. Fistarol and P. H. Itin , “Diagnosis and Treatment of Lichen Sclerosus: An Update,” American Journal of Clinical Dermatology 14, no. 1 (2013): 27–47, 10.1007/s40257-012-0006-4. 23329078 PMC3691475

[30] A. Lee , J. Bradford , and G. Fischer , “Long‐Term Management of Adult Vulvar Lichen Sclerosus: A Prospective Cohort Study of 507 Women,” JAMA Dermatology 151, no. 10 (2015): 1061–1067, 10.1001/jamadermatol.2015.0643. 26070005

[31] S. M. Neill , F. M. Lewis , F. M. Tatnall , and N. H. Cox , British Association of Dermatologists ., “British Association of Dermatologists' Guidelines for the Management of Lichen Sclerosus 2010,” British Journal of Dermatology 163, no. 4 (2010): 672–682, 10.1111/j.1365-2133.2010.09997.x. 20854400

[32] S. M. Cooper , X. H. Gao , J. J. Powell , and F. Wojnarowska , “Does Treatment of Vulvar Lichen Sclerosus Influence Its Prognosis?,” Archives of Dermatology 140, no. 6 (2004): 702–706, 10.1001/archderm.140.6.702. 15210461

[33] N. Oyama , I. Chan , S. M. Neill , et al., “Autoantibodies to Extracellular Matrix Protein 1 in Lichen Sclerosus,” The Lancet 362, no. 9378 (2003): 118–123, 10.1016/S0140-6736(03)13863-9. 12867112

[34] J. A. Carlson , R. Ambros , J. Malfetano , et al., “Vulvar Lichen Sclerosus and Squamous Cell Carcinoma: A Cohort, Case Control, and Investigational Study With Historical Perspective; Implications for Chronic Inflammation and Sclerosis in the Development of Neoplasia,” Human Pathology 29, no. 9 (1998): 932–948, 10.1016/s0046-8177(98)90198-8. 9744309

[35] J. M. Pugliese , A. F. Morey , and A. C. Peterson , “Lichen Sclerosus: Review of the Literature and Current Recommendations for Management,” Journal of Urology 178, no. 6 (2007): 2268–2276, 10.1016/j.juro.2007.08.024. 17936829

[36] A. Lis‐Święty , K. Mierzwińska , K. Wodok‐Wieczorek , M. Widuchowska , and L. Brzezińska‐Wcisło , “Co‐Existence of Lichen Sclerosus and Localized Scleroderma in Female Monozygotic Twins,” Journal of Pediatric and Adolescent Gynecology 27, no. 6 (2014): e133–e136, 10.1016/j.jpag.2013.11.010. 24841519

[37] G. L. Liu , F. L. Cao , M. Y. Zhao , J. Shi , and S. H. Liu , “Associations Between HLA‐A\B\DRB1 Polymorphisms and Risks of Vulvar Lichen Sclerosus or Squamous Cell Hyperplasia of the Vulva,” Genetics and Molecular Research 14, no. 4 (2015): 15962–15971, 10.4238/2015.December.7.8. 26662388

[38] S. Aidé , F. R. Lattario , G. Almeida , I. C. do Val , and M. da Costa Carvalho , “Epstein‐Barr Virus and Human Papillomavirus Infection in Vulvar Lichen Sclerosus,” Journal of Lower Genital Tract Disease 14, no. 4 (2010): 319–322, 10.1097/LGT.0b013e3181d734f1. 20885159

[39] A. Terlou , L. A. M. Santegoets , W. I. van der Meijden , et al., “An Autoimmune Phenotype in Vulvar Lichen Sclerosus and Lichen Planus: A Th1 Response and High Levels of microRNA‐155,” Journal of Investigative Dermatology 132, no. 3 Pt 1 (2012): 658–666, 10.1038/jid.2011.369. 22113482

[40] L. Wang , J. L. Yi , H. Y. Chen , P. L. Wang , and Y. L. Shen , “Level of Foxp3, DNMTs, Methylation of Foxp3 Promoter Region, and CD4+ CD25+ CD127low Regulatory T Cells in Vulvar Lichen Sclerosus,” The Kaohsiung Journal of Medical Sciences 37, no. 6 (2021): 520–527, 10.1002/kjm2.12356. 33438816 PMC11896417

[41] C. S. Sander , I. Ali , D. Dean , J. J. Thiele , and F. Wojnarowska , “Oxidative Stress Is Implicated in the Pathogenesis of Lichen Sclerosus,” British Journal of Dermatology 151, no. 3 (2004): 627–635, 10.1111/j.1365-2133.2004.06142.x. 15377350

[42] H. K. Haefner , K. C. Welch , A. M. Rolston , et al., “Genomic Profiling of Vulvar Lichen Sclerosus Patients Shows Possible Pathogenetic Disease Mechanisms,” Journal of lower genital tract disease 23, no. 3 (2019): 214–219, 10.1097/LGT.0000000000000482. 31232912

[43] G. Kirtschig , K. Becker , A. Günthert , et al., “Evidence‐Based (S3) Guideline on (Anogenital) Lichen Sclerosus,” Journal of the European Academy of Dermatology and Venereology 29, no. 10 (2015): e1–e43, 10.1111/jdv.13136. 26202852

[44] T. H. Wong , C. A. Morton , N. Collier , et al., “British Association of Dermatologists and British Photodermatology Group Guidelines for Topical Photodynamic Therapy 2018,” British Journal of Dermatology 180, no. 4 (2019): 730–739, 10.1111/bjd.17309. 30506819

[45] A. Niinimäki , M. Kallioinen , and A. Oikarinen , “Etretinate Reduces Connective Tissue Degeneration in Lichen Sclerosus Et Atrophicus,” Acta Dermato‐Venereologica 69, no. 5 (1989): 439–442. 2572115

[46] A. Cuellar‐Barboza , A. M. Bashyam , R. I. Ghamrawi , D. Aickara , S. R. Feldman , and R. O. Pichardo , “Methotrexate for the Treatment of Recalcitrant Genital and Extragenital Lichen Sclerosus: A Retrospective Series,” Dermatologic Therapy 33, no. 4 (2020): e13473, 10.1111/dth.13473. 32347617

[47] S. Terras , T. Gambichler , R. K. C. Moritz , M. Stücker , and A. Kreuter , “UV‐A1 Phototherapy Vs Clobetasol Propionate, 0.05%, in the Treatment of Vulvar Lichen Sclerosus: A Randomized Clinical Trial,” JAMA Dermatology 150, no. 6 (2014): 621–627, 10.1001/jamadermatol.2013.7733. 24696010

[48] E. Sotiriou , D. Panagiotidou , and D. Ioannidis , “An Open Trial of 5‐aminolevulinic Acid Photodynamic Therapy for Vulvar Lichen Sclerosus,” European Journal of Obstetrics & Gynecology and Reproductive Biology 141, no. 2 (2008): 187–188, 10.1016/j.ejogrb.2008.07.027. 18778886

[49] J. G. Hobson , S. F. Ibrahim , and M. G. Mercurio , “Recalcitrant Vulvar Lichen Sclerosus Treated With Erbium YAG Laser,” JAMA Dermatology 155, no. 2 (2019): 254–256, 10.1001/jamadermatol.2018.4461. 30540338

[50] A. D'Antuono , S. Bellavista , F. Negosanti , S. Zauli , E. Baldi , and A. Patrizi , “Dermasilk Briefs in Vulvar Lichen Sclerosus: An Adjuvant Tool,” Journal of lower genital tract disease 15, no. 4 (2011): 287–291, 10.1097/LGT.0b013e31821380a0. 21959572

[51] M. Corazza , A. Borghi , S. Minghetti , G. Toni , and A. Virgili , “Clobetasol Propionate vs. Mometasone Furoate in 1‐Year Proactive Maintenance Therapy of Vulvar Lichen Sclerosus: Results From a Comparative Trial,” Journal of the European Academy of Dermatology and Venereology 30, no. 6 (2016): 956–961, 10.1111/jdv.13166. 25904498

[52] E. Bulbul Baskan , H. Turan , S. Tunali , S. C. Toker , and H. Saricaoglu , “Open‐Label Trial of Cyclosporine for Vulvar Lichen Sclerosus,” Journal of the American Academy of Dermatology 57, no. 2 (2007): 276–278, 10.1016/j.jaad.2007.03.006. 17442452

[53] N. Manuelpillai , H. Saunders , and E. Veysey , “Management of Severe Vulval Lichen Sclerosus With Adalimumab,” Australasian Journal of Dermatology 63, no. 2 (2022): 248–250, 10.1111/ajd.13814. 35262179

[54] F. Behnia‐Willison , N. R. Pour , B. Mohamadi , et al., “Use of Platelet‐Rich Plasma for Vulvovaginal Autoimmune Conditions Like Lichen Sclerosus,” Plastic and Reconstructive Surgery ‐ Global Open 4, no. 11 (2016): e1124, 10.1097/GOX.0000000000001124. 27975027 PMC5142493

[55] A. Chiesa‐Vottero , P. M. Dvoretsky , and W. R. Hart , “Histopathologic Study of Thin Vulvar Squamous Cell Carcinomas and Associated Cutaneous Lesions: A Correlative Study of 48 Tumors in 44 Patients With Analysis of Adjacent Vulvar Intraepithelial Neoplasia Types and Lichen Sclerosus,” The American Journal of Surgical Pathology 30, no. 3 (2006): 310–318, 10.1097/01.pas.0000180444.71775.1a. 16538050

[56] J. L. Bercaw‐Pratt , L. A. Boardman , and J. S. Simms‐Cendan , “Clinical Recommendation: Pediatric Lichen Sclerosus,” Journal of Pediatric and Adolescent Gynecology 27, no. 2 (2014): 111–116, 10.1016/j.jpag.2013.11.004. 24602304

[57] A. Sergeant , N. Vernall , L. J. Mackintosh , P. McHenry , and J. A. Leman , “Squamous Cell Carcinoma Arising in Extragenital Lichen Sclerosus,” Clinical and Experimental Dermatology 34, no. 7 (2009): e278–e279, 10.1111/j.1365-2230.2008.03195.x. 19438563

[58] E. Delpero , A. Sriharan , and A. Selk , “Steroid Phobia in Patients With Vulvar Lichen Sclerosus,” Journal of Lower Genital Tract Disease 27, no. 3 (2023): 286–290, 10.1097/LGT.0000000000000753. 37285240

[59] A. Ranum and D. R. Pearson , “The Impact of Genital Lichen Sclerosus and Lichen Planus on Quality of Life: A Review,” International Journal of Women's Dermatology 8, no. 3 (2022): e042, 10.1097/JW9.0000000000000042. PMC9387966 36000015

[60] R. B. Saunderson , V. Harris , R. Yeh , K. A. Mallitt , and G. Fischer , “Vulvar Quality of Life Index (VQLI) ‐ A Simple Tool to Measure Quality of Life in Patients With Vulvar Disease,” Australasian Journal of Dermatology 61, no. 2 (2020): 152–157, 10.1111/ajd.13235. 31984477

[61] X. Xie and K. Wu , “Advances in the Pathogenesis of Vulvar Lichen Sclerosus,” Molecular Biology Reports 51, no. 1 (2024): 396, 10.1007/s11033-024-09318-7. 38453810

[62] J. Zhang , R. Zhao , Y. Huang , et al., “The Application of Tranexamic Acid in Joint Arthroplasty: A 20‐Year Bibliometric Analysis,” Frontiers in Public Health 10 (2022): 1013461, 10.3389/fpubh.2022.1013461. 36388346 PMC9664086

