# Supplementary material for: Trends and Developments in Vulvar Lichen Sclerosus Research
Source: Health Sci Rep. 2025 Jul 9;8(7):e71007. doi: 10.1002/hsr2.71007 (PMC12239157; doi:10.1002/hsr2.71007)
Supplement: Supplementary file 1 — Table S1. [file HSR2-8-e71007-s001.pdf]

Supplemental Table 1. Summary of data source and selection

Category	Specific Standard Requirements
Research database	Web of Science Core Collection
Citation indexes	SCI-E,SSCI
Searching period	January 1st 1994 to December 31st 2023
Language	“English”
Searching Keywords	“LICHEN SCLEROSIS,LICHEN SCLEROSUS,LICHEN SCLEROSUS ET ATROPHICUS, VULVAR DERMATOSES,<br>VULVAR LICHEN SCLEROSUS,<br>VULVAL LICHEN SCLEROSUS”
Document types	“Articles”
Data extraction	Export with full records and cited references in plain text format
Total number	1,698
